# Management of patients with conversion disorder

**DOI:** 10.1192/j.eurpsy.2022.1203

**Published:** 2022-09-01

**Authors:** I. Baati, M. Ben Abdallah, A. Arous, S. Hentati, F. Guermazi, J. Jdidi, J. Masmoudi

**Affiliations:** 1 CHU Hedi CHaker hospital Sfax Tunisia, Department Of Psychiatry (a), Sfax, Tunisia; 2 CHU Hedi CHaker hospital Sfax Tunisia, Department Of Community Health And Epidemiology, Sfax, Tunisia

**Keywords:** Conversion Disorder, Primary Care Physician, management

## Abstract

**Introduction:**

Conversion disorder (CD) is largely managed by primary care physician. A good knowledge of this disorder and a mastery of adequate therapeutic means will allow patients to recover promptly and reduce recurrences.

**Objectives:**

To evaluate the management of CD by primary care physicians.

**Methods:**

This cross-sectional and descriptive study involved 90 primary care physicians in the region of Sfax (Tunisia). We submitted a self-administered anonymous questionnaire to physicians to explore their practice towards patients with CD.

**Results:**

Among the 90 doctors contacted, 54 (60%) responded to our questionnaire. Their age ranged from 25 to 70 years, with a median of 41 years. The sex ratio was 0.92. The average number of years of practice was 15 years (SD = 9.7). Half of the physicians reported that the consultation of a patient with CD lasted between 15 and 30 minutes. Faced with a first episode of CD, 61.1% of the doctors decided to treat the patient alone and 18.5% preferred to take the advice of a psychiatrist. In the case of a recurrence, 59.2% chose to refer the patient immediately to a psychiatrist. The use of pharmacological treatment was indicated by 64.8% of participants. Half of the doctors stated that they had difficulties in managing patients with CD.

**Conclusions:**

According to our results, the management of CD by primary care physicians remained restrictive and difficult. It is therefore necessary to encourage primary care physicians to express the difficulties they encounter and to turn to their psychiatric colleagues for help.

**Disclosure:**

No significant relationships.

